# Addenbrooke's Hospital, Cambridge: Quarterly Court

**Published:** 1918-02-23

**Authors:** 


### ADDENBROOKE'S HOSPITAL, CAMBRIDGE: Quarterly Court.

The Effect of Increased Prices.

The Mayor of Cambridge, the Rev. E. C. Pearce, D.D., Master of Corpus Christi College, presided at the General Quarterly Court held on February 18.

The report upon the work of the past quarter to December 31, 1917, showed that the expenditure exceeded that of the corresponding quarter of 1916 by about £1,785. This increase was explained by the increase in cost of (1) provisions, (2) surgical dressings, (3) salaries and wages, (4) domestic and establishment charges, and (5) additional patients.

The increase of about £550 in provisions was accounted for by an increase in price of about 6d. per lb., plus the cost of a stock of tinned meat bought forward. Fish had increased by 5d. per lb., and tinned fish had been bought forward to prepare for contingencies, such having to be used for soldiers' breakfasts instead of eggs or bacon which are difficult to obtain. Milk had increased by 4½d. per gallon, and the price of all groceries had been steadily rising.

The increase in surgical dressings was principally due to the purchase in advance at advantageous prices of large stocks of certain dressings to last for a year. The increase on domestic and establishment charges was due to the remaking and recovering of over 100 hair mattresses, the purchase of a reserve stock of 100 tons of slack and 100 tons of house coal, the purchase of stocks of hardware, crockery, soap, and other cleansing materials. The increase in salaries and wages was due to higher rates of salaries paid to resident medical officers, pharmacy staff, laundry and domestic staff, etc.

There were decreases as follows, amounting to £241: Bacon, £45; eggs, £22; bread, £23; malt liquors, £47; mineral waters, £22; drugs, £58; and printing, £24, thereby reducing the net increase to about £1,550.

The Vice-Chairman explained that the prudence of the system of buying was shown by one instance where by the purchase of flannelette and eye muslin when opportunity offered the hospital was saved on an actual expenditure of about £100 at least £80.

Contested Will: The Hospital as Residuary Legatee.

The Chairman of the General Committee drew the attention of the Governors to the will of Mr. Bailey, late of Cambridge, which had been contested, and to the fact that the Judge had given judgment in favour of the will, so that as therein provided the hospital would, after certain small bequests and a life interest, be entitled to the residue as residuary legatees to a sum estimated to be about £8,000 to £10,000.

Resignation of Secretary-Superintendent: The Committee's Tribute.

The General Committee reported having received the resignation of the secretary-superintendent, Mr. Richard J. Coles, upon his appointment to the secretaryship of King's College Hospital. The Chairman, the Rev. J. B. Lock, said the General Committee received Mr. Coles' resignation with very great regret. Mr. Coles was appointed to the secretary-superintendentship in 1904, when that office was created, and during the thirteen years he had served the hospital he had carried out his duties with marked zeal and energy, and his never-failing tact and discretion in the administration of the hospital had been shown very conspicuously. They heartily congratulated Mr. Coles and wished him every success.

The Chairman proposed that the Court of Governors endorse the opinion expressed by the General Committee, and tender to Mr. Coles their appreciation of his valuable services to the hospital for the past thirteen years and their good wishes in his new and important post. Alderman Stoll seconded, and the proposition was carried.

